# Insights into the role of differential gene expression on the ecological adaptation of the snail *Littorina saxatilis*

**DOI:** 10.1186/1471-2148-10-356

**Published:** 2010-11-18

**Authors:** Mónica Martínez-Fernández, Louis Bernatchez, Emilio Rolán-Alvarez, Humberto Quesada

**Affiliations:** 1 Departamento de Bioquímica, Genética e Inmunología. Facultad de Biología. Campus As Lagoas-Marcosende. Universidad de Vigo, 36310 Vigo. Spain; 2 IBIS (Insitut de Biologie Intégrative et des Systèmes), Pavillon Charles-Eugène-Marchand. 1030, Avenue de la Médecine. Université Laval. Québec (Québec) G1V 0A6. Canada

## Abstract

**Background:**

In the past 40 years, there has been increasing acceptance that variation in levels of gene expression represents a major source of evolutionary novelty. Gene expression divergence is therefore likely to be involved in the emergence of incipient species, namely, in a context of adaptive radiation. In this study, a genome-wide expression profiling approach (cDNA-AFLP), validated by quantitative real-time polymerase chain reaction (qPCR) were used to get insights into the role of differential gene expression on the ecological adaptation of the marine snail *Littorina saxatilis*. This gastropod displays two sympatric ecotypes (RB and SU) which are becoming one of the best studied systems for ecological speciation.

**Results:**

Among the 99 transcripts shared between ecotypes, 12.12% showed significant differential expression. At least 4% of these transcripts still displayed significant differences after correction for multiple tests, highlighting that gene expression can differ considerably between subpopulations adapted to alternative habitats in the face of gene flow. One of the transcripts identified was Cytochrome c Oxidase subunit I (COI). In addition, 6 possible reference genes were validated to normalize and confirm this result using qPCR. α-Tubulin and histone H3.3 showed the more stable expression levels, being therefore chosen as the best option for normalization. The qPCR analysis confirmed a higher COI expression in SU individuals.

**Conclusions:**

At least 4% of the transcriptome studied is being differentially expressed between ecotypes living in alternative habitats, even when gene flow is still substantial between ecotypes. We could identify a candidate transcript of such ecotype differentiation: Cytochrome c Oxidase Subunit I (COI), a mitochondrial gene involved in energy metabolism. Quantitative PCR was used to confirm the differences found in COI and its over-expression in the SU ecotype. Interestingly, COI is involved in the oxidative phosphorylation, suggesting an enhanced mitochondrial gene expression (or increased number of mitochondria) to improve energy supply in the ecotype subjected to the strongest wave action.

## Background

Unravelling processes that underlie population divergence is a crucial step towards elucidating the origin and maintenance of biodiversity [1], and towards understanding the genetic basis of speciation, which is one of the most fundamental goals in evolutionary genetics [2]. However, there is still much to be learned about how divergent populations adapt to different environments under the effect of natural selection, which ultimately may evolve into biological species [3]. The new "omics" technologies, despite being very young, can contribute to this since they have taken up a very important position in the biological scientific landscape during the last decade [4]. Actually, in the current postgenomic era, researches can now not only determine the genome of the organisms of interest, but also the transcriptome, the proteome and their relationships and characteristics even over time. Genome-wide surveys of patterns of gene expression, for example, can help by revealing the functional importance of correlations between gene expression and the development of a phenotype [5]. Moreover, evolutionary shifts in gene expression profiles could be used to explore genetic targets involved in local adaptation and ecological speciation [6].

The development of microarray technology has revolutionized the study of expression profiles since scientists can study tens of thousands of genes at once. However, its utility has been restricted to organisms with extensive cDNA or genomic DNA sequence data available and, of course, any key question in ecology and evolutionary biology cannot be solely addressed using model organisms [7]. A useful alternative technique for transcriptome profiling is cDNA-amplified fragment length polymorphism analysis (cDNA-AFLP), a modification of the original genomic DNA-AFLP [8,9], by means of which accurate gene expression profiles can be determined by quantitative analysis of the peak (transcript) intensities generated [10,11]. It represents a reliable technique due to the highly stringent PCR conditions, and no prior sequence information is required. Moreover, the mean variance of data is similar for cDNA-AFLP and microarray hybridization [12]. It also has the advantage over other gel-based methods that each selective primer combination displays a different subset of cDNAs, thus facilitating experimental replication of genome-wide expression patterns [13]. The cDNA-AFLP technique has been broadly used in plants to identify genes involved in defence, resistance, sterility, adaptation or acclimation to contrasting environments, and to study the heritability of the intensity polymorphisms [13-22].

On the other hand, quantitative PCR (qPCR) is currently one of the most powerful and sensitive techniques for analyzing gene expression, being often used for validating output data produced by micro-and macro-arrays or other open expression systems. In order to avoid experimental errors arising from variation in the quantity and integrity of the RNA template, as well as the efficiency of the cDNA synthesis and PCR amplification, a normalization step is an essential pre-requisite. Among the several proposed methods [23,24], internal control genes (reference genes), formerly called housekeeping genes, are most commonly used to normalize qPCR and to reduce possible errors generated in the quantification of gene expression [25]. Such genes are supposed to be constitutively expressed at a constant level under various experimental conditions, in different tissues types and developmental stages. However, the assumption of a stable expression in every cell and tissue has proven false by a growing number of studies [26-29]. In fact, all genes seem to be regulated under some conditions, such that there is no single universal reference gene with a constant expression in all tissues [29-34]. Therefore, a careful validation of the usefulness of potential reference genes is essential. To date and under our knowledge, only a limited number of reference genes have been identified in gastropods and none of them has been validated [35-42]. In the present study, 6 novel candidate reference genes suitable for gene expression normalization were identified and validated in the marine snail *Littorina saxatilis *(Olivi, 1792) (Gastropoda, Prosobranchia).

Therefore, here we will use the cDNA-AFLP technique and qPCR to compare the expression profiles between two ecotypes of *Littorina saxatilis *involved in an incomplete sympatric ecological speciation process [43-45], in order to provide insights into the role of differential gene expression on ecological adaptation.

The marine snail *Littorina saxatilis *has separate sexes, internal fertilization, and a brood pouch with non-planktonic shelled embryos. In the exposed Galician coast (NO Spain), two well differentiated ecotypes are adapted to different shore levels and habitats [45,46]. The RB ecotype (Ridged and Banded) lives on barnacles in the upper shore. This ecotype displays a larger and more robust shell to resist the attack from predators such as crabs, and a smaller shell aperture in order to reduce the desiccation due to high sunshine exposure [47-49]. The SU ecotype (Smooth and Unbanded) is found at the lower shore living on mussels. This ecotype shows a smaller and thinner shell with a wider shell aperture to allocate a relatively larger muscular foot providing a higher ability to avoid the dislodgment caused by the heavy wave action [47-51]. Both ecotypes coexist in an intermediate habitat at the middle shore, where RB and SU individuals meet and occasionally mate, showing an effectively sympatric ecotype distribution, and a partial pre-zygotic isolation barrier (i.e. assortative mating; see [45]).

Gene flow between ecotypes is only slightly restricted (8-20%) compared to the level of gene flow among different populations within ecotypes [45,52,53]; therefore the polymorphism observed is due to strong divergent natural selection acting across the environmental gradient [47,49,52]. Thus, these two *Littorina saxatilis *ecotypes represent an interesting system to study the genetic basis of adaptive ecological divergence at the transcriptional level in marine species. This work builds upon earlier genome-wide studies showing differences in DNA sequence and protein expression between these ecotypes in the same population [52,54], allowing a more global picture of the ecological adaptation process through the integration of different "omics" resources.

## Results

### cDNA-AFLP analysis

A total of 16 biological replicates were sampled per ecotype across 2 different transects (therefore, 8 replicates by transect). Each biological replicate included a pool of 10 adult snails (5 males and 5 females). After total RNA extraction, the quality of the RNA was confirmed spectrophotometrically by the A260/A280 absorbance ratio that ranged from 1.92 to 2.06, indicating a high purity for the RNA extractions. For the cDNA-AFLP analysis, each sample was run twice (technical replicates). Approximately, a total of 2,800 transcripts (75-500 bp long) were obtained from 12 primer combinations using the restriction enzyme pair *Mse*I-*Taq*I, with an average of 98.11 ± 1.18 transcripts per primer combination. However, only 99 transcripts were shared in at least 90% of the biological and technical replicates, and therefore considered in the comparison between ecotypes.

The coincidence for the presence/absence data was compared by a coefficient of similarity, specifically the "simple-matching coefficient" [55]. Thus, the coefficient across samples was 0.88 (in all cases the coefficients showed a *P *< 0.05). Regarding the quantitative data, the averaged Pearson correlation coefficient across samples was used to measure the reproducibility, showing the value of 0.73 (again *P *< 0.05 always), which increased to 0.82 (*P *< 0.05) when only the transcripts with significant expression differences were taken into account.

Quantitative transcript differences between ecotypes were assessed using a three-way ANOVA (ecotype and transect as fixed factors and the biological replicate nested with interaction as the third factor) on the normalized intensity data (99 transcripts) obtaining 12 transcripts (12.12%) with different level of expression between ecotypes (Table 1), 9 of them characterized by higher level of expression in the SU ecotype. This percentage was reduced to 4% (4 significant transcripts) when adjustment for multiple tests was performed. Results do not change significantly when using a less restrictive cut-off criterion to select the peaks included in the quantitative analysis. When considering those peaks present in at least 80% of replicates (241 transcripts), 12.4% displayed expression differences between ecotypes after the three-way ANOVA, and 4.6% after the SGoF (Sequential Goodness of Fit test) correction. Thus, our 90% cut-off for peak presence among replicates can be considered as rather conservative, although we preferred to be stricter and more restrictive at the expense of having fewer but more reliable transcripts.

**Table 1:** Transcripts analyzed by a three-way nested ANOVA that showed significant expression differences between ecotypes.

Transcripts	*F*_Ecotype_	*F*_Transect_	*F*_E × T_	*F*_Specimen(E × T)_	RB (average ± ES)	SU (average ± ES)	*F*_randomization_
1^#^	8.93**	0.19	0.12	6.07**	1057 ± 225.9	2317 ± 341.0	9.48**
10	6.87*	0.012	0.24	12.38***	715 ± 161.9	258 ± 43.1	7.41*
29	38.70***	0.62	2.40	0.53	304 ± 94.9	649 ± 100.8	6.20*
41	6.49*	1.53	0.002	1.41	1023 ± 335.6	4381 ± 411.3	39.94***
57	10.71**	0.43	0.11	2.98*	1289 ± 343.1	3038 ± 435.5	11.13***
63	5.57*	1.04	0.23	6.14**	1441 ± 479.3	3388 ± 674.0	5.53*
64	11.01**	1.69	0.07	9.67***	579 ± 144.6	2130 ± 458.6	10.40**
73	5.98*	0.46	0.09	5.77**	652 ± 175.9	1443 ± 264.4	6.20*
76^#^	8.13*	0.01	0.05	24.34***	2342 ± 652.7	441 ± 77.9	7.25*
78	7.22*	0.63	0.07	5.52**	1108 ± 230.3	402 ± 156.9	5.82*
85	6.43*	0.01	0.14	3.22*	464 ± 54.1	1605 ± 429.9	6.92**
96	6.15*	2.14	0.87	2.43	391 ± 105.9	1117 ± 303.7	4.47*

*P ≤ 0.05; **P ≤ 0.01; ***P ≤ 0.001

# loci showing significant expression between sexes of the RB ecotype [11].

We represent the *F *value for the factors ecotype, transect, interaction, and nested considering the biological replicates. In RB and SU, the means± standard errors of the RFU (relative fluorescence units) are shown. The randomization ANOVA (*F*_randomization_) was analyzed on the mean trancript intensity across technical replicates. Underlined probabilities were still significant after multitest correction by SGoF [67].

None of the transcripts with significant differences in gene expression between ecotypes were significant by the transect factor nor by the interaction. Thus, differences in gene expression for these transcripts were related mainly with the ecotype, making them potential candidate loci. All the 12 significant cases between ecotypes were confirmed by a randomization ANOVA using the averaged intensity data from the two technical replicates. Here, 4 remained significant after multitest correction (4%), and 2 of them (numbers 1 and 57) matched with those that were also statistically significant after multitest correction in the three-way ANOVA (Table 1). Interestingly, 2 of these transcripts (numbers 1 and 76) matched with those that have been shown to display significant expression differences between sexes in a previous study performed in this same population [11].

**Figure 1:**
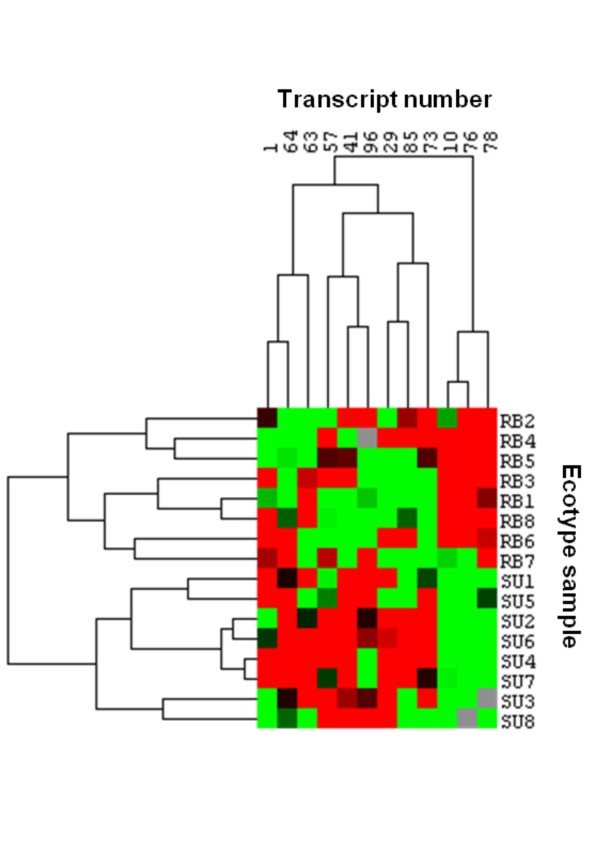
**Hierarchical clustering of the transcripts based on their expression profiles (using the averaged values from the technical replicates)**. Rows represent the pools of individuals from each ecotype and columns represent the transcripts. Red indicates enhanced expression while green reflects decreased expression. All individuals were correctly clustered by their ecotype.

Quantitative analysis and hierarchical clustering of the expression patterns revealed two well differentiated groups of transcripts behaving differentially between the two ecotypes (Figure 1). Pools of individuals were correctly clustered by ecotype based on their expression profiles, showing a clear distinction between genes that were up or down regulated in the RB ecotype (or the opposite in the SU ecotype).

### Sequencing and identification of transcripts

Six out of 12 transcripts with differential expression between ecotypes showed clear size differences with respect to the other fragments separated in the same AFLP run, making easier their isolation for sequencing purposes. These were therefore isolated and cloned. PCR products obtained from several bacterial colonies containing the insert with the right size were sequenced. All inserts of a particular transcript size were found to be of identical sequence, except for a single heterozygous site in transcript 78 (table 2). Thus, differentially expressed transcripts were not due to increased signal from the co-migration of non-homologous transcripts at a given size. The sequences obtained from the 6 isolated transcripts were compared against those present in the databases. After BLAST and TBLAST searches, 2 transcripts did not show any hit (64 and 85 in table 2), whereas transcript number 1 showed significant hits (Table 2). This transcript was identified as a known protein of *Littorina saxatilis*: the Cytochrome c Oxidase subunit I (COI). This gene seems to be over-expressed in the SU ecotype. Interestingly, this same COI transcript also displayed a differential gene expression between sexes, being higher expressed in males than in females [11].

**Table 2:** Length of the sequenced candidate transcripts, numbered following table 1.

Transcript number	Length (bp)	E value	Alignment	Species	Function	Reference
1	71	2 × 10^-24^	98%	*Littorina saxatilis*	Cytochrome Oxidase Subunit 1	emb|AJ132137.1
10	73	8.7 × 10^-2^	61%	*Danio rerio *(Zebrafish)	Specific membrane neuromodulator (similar to Galanine)	emb|AL831791.5
63	95	4 × 10^-3^	48%	*Caenorhabditis briggae*	Unknown Protein	XM|001666586.1
64	114	N/A				
78	186	4.7 × 10^-2^	51%	*Danio rerio *(Zebrafish)	Cytoplasmatic anchor protein (p53-associated parkin protein)	XM|001666586.1
85	82	N/A				

Only hits with an e-value below 10^-2 ^have been listed.

N/A: not applicable because these transcripts did not show any hit.

### qPCR: gene expression stability analyses and ranking of selected reference genes

In order to validate this result, one of our main interests focused on elucidating which genes could be used as reference genes for qPCR. We selected 6 potential reference genes, and designed primer pairs for each one (Table 3). These genes were chosen in this species because they were commonly used for other organisms or because their functional description indicated they might be useful candidate reference genes. Within our means, special attention was paid to selecting genes that could belong to different functional classes, which significantly reduces the likelihood that genes might be co-regulated. All of them produced a single peak in the melting curve analyses performed following the qPCR. No amplification was detectable in the absence of template.

**Table 3:** List of primers and reference genes (names, function and amplicon size) under investigation. The primers for the gene of interest (COI) are also included.

Gene	Forward primer	Reverse Primer	Function	Product size (bp)
18S	5'-GGTTTTCGGAACACGAGGTA-3'	5'-TGGCATCGTTTATGGTCAGA-3'	Small ribosomal subunit	200
α Tubulin	5'-CCATACCCTTCACCGACGTA-3'	5'-AGGTGGGCATCAACTACCAG-3'	Structural constituent of cytoskeleton	190
Histone H3.3	5'-AGAGTGCTCCCTCAACTGGA-3'	5'-GTCCTCAAAGAGACCCACCA-3'	To mark active chromatin (nucleosome structure)	194
Elongation Factor 1α	5'-GCCCTTGAACCACTTCATGT-3'	5'-ATCATCGGCGTCAACAAGAT-3'	Translational elongation	195
Elongation Factor 2	5'-ACGCATGTTCTCCTCACACA-3'	5'-CGCTACCTGGTGGACAACTT-3'	Translational elongation	198
Calmodulin	5'-CACCGTTCGTTTCATCCATA-3'	5'-GTTCTGTCCCAGCGACCTC-3'	Calcium-binding protein	182

COI	5'-GGGGGAGGAGACCCTATTCT-3'	5'-ATGGTGGGCCCATACAATAA-3'	Energy production (electron transport chain)	204

The expression stability of the set of candidate reference genes was examined by geNorm software, which calculates, for each gene, a measure of its expression stability (M) based on the average pairwise variation between all genes tested. Stepwise exclusion of the least stable gene allowed the genes to be ranked according to their M value (the lower the M value, the higher the gene's expression) [31]. All genes had a value below the geNorm threshold of 1.5, being theoretically good reference genes (18S = 0.65; calmodulin = 0.49; EF1 = 0.37; EF2 = 0.31; Histone and Tubulin = 0.29).

**Figure 2:**
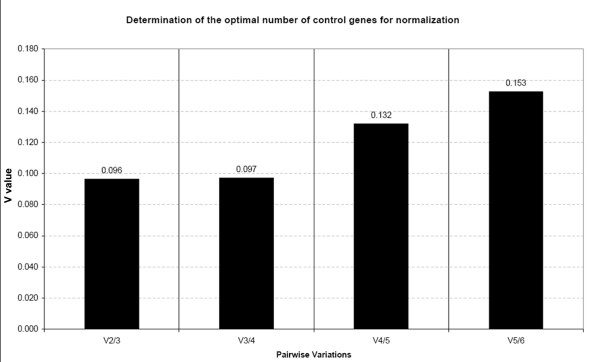
**Determination of the optimal number of reference genes for data normalization**. Bar values indicate the magnitude of change in the normalization factor after the inclusion of an additional reference gene. GeNorm authors' suggest that V > 0.15 should be considered as the threshold to include an extra reference gene into the assay.

**Figure 3:**
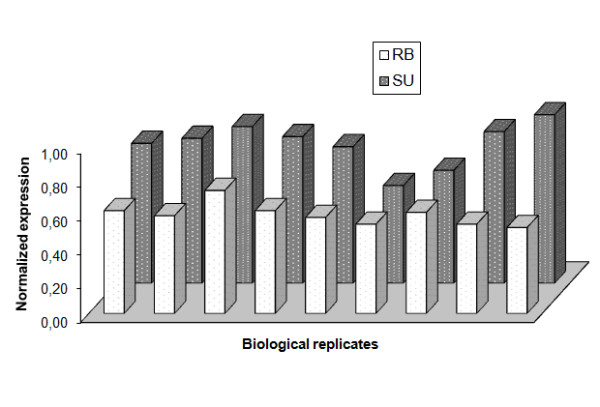
**Normalized expression of COI for each ecotype after qBASE analysis**. In all the biological replicates, pooled individuals of SU ecotype showed a higher expression of COI.

The geNorm program, in addition to the gene stability measure M, computes a normalization factor (NF) and assesses the optimal number of reference genes required for normalization. The pairwise variation (Vn/Vn+1) between two sequential normalization factors (NFn and NFn+1) are used to determine the necessity of adding the next more stable control gene for reliable normalization [31]. Pairwise variations were calculated using geNorm for each data set to determine the optimal number of internal control genes for normalization (Figure 2). As reported by Vandensompele et al. [31], a threshold value of 0.15 for this pairwise variation (Vn/Vn+1) was adopted. Since the V2/3 value (pairwise variation between using 2 or 3 reference genes) was 0.096 (Figure 3) while the V3/4 value was very similar (0.097), it can be concluded that normalization using only the 2 most stable reference genes (histone together with tubulin) would be sufficient.

### COI expression

In order to validate the differential COI expression detected with the cDNA-AFLP technique, primers were designed to amplify COI. The PCR product was isolated and sequenced, confirming its identity (Table 3). Then, qPCR analyses were carried out using 9 new biological replicates per ecotype (again, each consisting of a pool of 5 males and 5 females). To determine the reproducibility of the qPCR method, these samples were analyzed in two consecutive days to obtain two technical replicates that were used to compare their raw Cq (quantification cycle) values for tubulin (r = 0.90; DF = 16; P ≤ 0.001, after 10,000 randomizations). Once a good reproducibility was obtained, the confirmation of the differential COI expression was assessed using the 9 biological replicates, and tubulin and histone as reference genes for the normalization. The raw Cq values obtained for COI were transformed to relative normalized quantities using the Cq values for histone and tubulin by the software qBase. After randomization, a significant differential gene expression was confirmed using one-way ANOVA (F = 24.97; P ≤ 0.0001 after 10,000 randomizations), confirming a higher expression in the SU ecotype (Figure 3), as showed in the previous cDNA-AFLP analyses.

## Discussion

Gene expression variation is widespread among individuals and taxa, has a heritable component, and it is subject to influence by natural selection and genetic drift [56,57]. Therefore, transcriptome analysis should provide insights into which genes are "important", besides being a common way of discovering differences in gene expression because regulation of gene activity occurs primarily at the transcription level [58]. In this study, we used a new variant of cDNA-AFLP technology to analyze differences in gene expression between two ecotypes of the marine snail *Littorina saxatilis*, considered an example of incomplete ecological and sympatric speciation. The experimental reproducibility obtained in this work for the cDNA-AFLP technique is in agreement with previous studies using this technology [11,59-61]. Since a pooling strategy has shown previously similar efficiency to the individual strategy for gene expression comparisons [62], we analyzed pools of 10 individuals (5 males and 5 females) with the aim of reducing individual effects, thus increasing the power to detect differences between ecotypes. We found that about 4% of the studied transcripts (12% without multitest adjustments) showed differences in expression between ecotypes. We note that our results would be conservative under a hypothetical scenario in which only one of the sexes contributed to expression differences between ecotypes, by averaging in each pool the input due to males and females.

Although the cDNA-AFLP technique is an end-point PCR technique, we note that a crucial characteristic of the AFLP technology is that it is semi-quantitative: the relative intensity of a PCR fragment band in an AFLP fingerprint is related to the original abundance of that fragment in the AFLP template [63,64]. Even though alternative quantification techniques such as real-time PCR can be several fold more sensitive, our results should be considered for this reason as conservative. This is reinforced by the fact that our conclusions are based on the existence of consistent profiling patterns across both biological and technical replicates. Thus, although small random differences at the start of the amplification may have a large effect on the final amount of PCR product, it is rather unlikely that these fluctuations can produce a repetitive systematic pattern across replicates. Indeed, the probability of getting such a pattern by chance is negligible, as indicated by the ANOVA analysis, even considering multiple testing.

Similarly, any putative technical or biological problem associated to this study was resolved by using two complementary types of controls: First, using both biological and technical replication and showing differences in the biological replicates despite of the detected technical noise. Second, gene expression differences between ecotypes were compared with those observed within ecotypes. So, any possible biases affecting our study should affect similarly to the within-and between-ecotype differentiation, while our results showed that significant transcript differences were observed exclusively between ecotypes.

The observed differences in gene expression could be heritable, caused by environmental effects linked to the different habitats associated to each ecotype, or result from a combination of environmental and heritable factors. Nevertheless, a recent study has shown that much of the variation in mRNA expression is related to genetic variation, and that only a minor part of this variation occurs in response to environmental changes [65]. Similarly, previous studies indicated that most of the protein expression and morphological differentiation observed between the ecotypes in the same population were not plastic [66,67]. Therefore, and even though it is obvious that environment has a role in gene expression, it seems reasonable to hypothesize that much of the variation found here could be at least primarily influenced by genetic factors, although the exact quantification of the extent of this genetic determination will require further experimental work comparing expression (transcriptomic) differences in wild and laboratory-reared snails.

Sorting or clustering genes into groups according to their expression patterns can provide a broad and interpretable overview of gene regulation [7]. Here, we carried out a cluster analysis in order to identify sets of genes with common expression between ecotypes. We could clearly distinguish two different groups of genes differentially expressed which allowed clustering all the pooled individuals by their ecotype (Figure 1). These two sets of genes could represent different functional groups involved in similar metabolic pathways or, at least, similarly regulated, thought a future confirmation would be needed.

Regarding the identification of the transcripts, most of the differentially expressed genes did not correspond to known sequences in the available databases. This could be due to the scarcity of gastropod sequences in public databases or, alternatively, these sequences may represent novel mRNAs. Another possibility is that they could correspond to the 3' untranslated region of the gene where the sequences are often less conserved than the sequences of protein-coding regions [68]. However, this possibility was reduced in the current study thanks to the modification applied to the original cDNA-AFLP protocol, in which the 3'-end tails close to the poly(A) tail were discarded (see Material and Methods).

When analyzing gene expression data by qPCR, stability of candidate reference genes and an appropriate method of normalization must be carefully evaluated. An ideal reference gene should be expressed at a constant level in each sample, but nowadays it is broadly accepted that the ideal and universal reference gene does not exist [29,69,70]. Unfortunately, for many organisms a large sequence dataset is not available, precluding the identification of genes whose transcripts are maintained at stable levels across the samples being surveyed [71]. In fact, this prior validation of reference genes remains uncommon in gastropods [35-42], or even in molluscs, though with exceptions [70]. To the best of our knowledge, the present study represents the first effort aimed at the identification and validation of reference genes for gene expression in a marine gastropod. The recently developed MIQE (the Minimum Information for publication of Quantitative real-time PCR Experiments) guidelines [72] suggest employing the geometric average of multiple reference genes and assessing gene stability with the support of validated mathematical models such as geNorm [31]. Even when there are few sequences available for *L. saxatilis*, we decided to discard β-actin gene, one of the most traditionally used in qPCR, for two main reasons: i) both ecotypes show differences in musculature (proportionally, SU ecotype has a bigger food muscle [54]); ii) it has proven to have an unstable expression in several organisms [73,74]. According to geNorm, the threshold M value for considering a gene to be unsuitable for data normalization is suggested to be ≥ 1.5 [31]. In this study, the 6 tested genes showed M values below 1.5, being possible good candidates to be used in the normalization. Based on this estimates, geNorm ranks the stability of the six genes in the following order: 18S < Calmodulin < EF1 < EF2 < Histone and Tubulin. Moreover, low values of the pair-wise variation V between two sequential normalization factors containing an increasing number of genes (Figure 2) showed it was unnecessary to include more than the two genes chosen by the geNorm software: histone and tubulin (M = 0.29). Since these two genes are involved in distinct biological processes and metabolic pathways (Table 3), have therefore a smaller chance of being co-regulated genes.

The analysis of qPCR profiles for the candidate gene Cytochrome c Oxidase subunit I (COI) confirmed the results obtained previously by cDNA-AFLP. This up-regulation of the COI in the SU ecotype could result both from an increased number of mitochondria in the SU ecotype and/or changes in transcription rate *per se*. Previous studies showed that higher levels of mtDNA gene products under physiological stimuli are primarily met by mtDNA replication (reviewed in [75]). Cytochrome c oxidase (COX) or Complex IV (EC 1.9.3.1), is a large transmembrane protein complex found in bacteria and in the mitochondrion. Specifically, subunit 1, like subunits 2 and 3, is a large and highly hydrophobic protein encoded in the mitochondrial genome [76]. COX is the last enzyme in the respiratory electron transport chain of mitochondria (or bacteria). It plays a fundamental role in energy production of aerobic cells, and also contributes to the storage of energy in the form of an electrochemical gradient that will be used by the oxidative phosphorylation system for synthesis of ATP [76]. Changes in the transcription level of genes involved in energy metabolism have been reported previously, and are especially interesting since they may influence important traits [57]. As COX activity can be modulated according to the energetic requirement of the cell, its increase in SU individuals could be related to a need of energy to avoid the dislodgement by the heavy wave action typical of their habitat. In fact, a very similar result was found in the same population at the proteome level [54], where enzymes related with the energetic metabolism (arginine kinase and fructose bisphosphate aldolase) were also up-regulated in the SU ecotype. Consequently, the provision of ATP and the control of its metabolism seem to be critical components of the general environmental stress response in all organisms, allowing them to respond with adaptive physiological changes while, at the same time, buffering the changing energy demands [7]. The SU individuals have to develop higher muscular effort than the other ecotype to be able to hold on the rocks while suffering the strong swell of their habitat [54]. Therefore, increasing the energetic metabolism could represent a possible adaptive physiological mechanism underlying differential muscular effort between both ecotypes. Future efforts aimed at determining whether this difference is due to environmental or genetic causes have to be made, for example studying its expression in individuals grown in lab conditions. Similar results have been found between sympatric species of lake whitefish and brook charr, where genes involved in energy metabolism emerged as prime candidates underlying their adaptive divergence [77,78]. Furthermore, and remarkably, the same transcript corresponding to the COI, together with the transcript 76 not identified, showed a higher expression in RB males *versus *RB females in a previous work [11]. A similar result was found in gene expression studies between sexes in *Drosophila spp*, where males showed a higher degree of variation in expression in genes associated with mitochondria and defence functions [79,80]. This is of particular interest given that sex-biased genes, especially those genes with male-biased expression, appear to evolve faster and, therefore, develop a higher divergence between species [79,81-84]. Taking into account that the sex dimorphism in size (males are smaller than females [45]) is much more pronounced in the SU ecotype than in the RB, the coincidence of genes participating in sexual and ecotype differentiation is plausible and raises the hypothesis that genes associated with the male reproductive function may contribute disproportionably to speciation, i.e. faster male theory [85,86].

A major challenge towards a comprehensive analysis of speciation processes is the integration of data from different "omics" resources and their interpretation. Even when we are very cautious and taking all the precautions due to the inherent differences caused by the distinct techniques, we could make a proxy of what is happening at three different biological levels. At genome level, both ecotypes differed around 3% in their genome by an AFLP scan [52], whereas the transcripts analyzed in the current study displayed 4% of gene expression differences. In general, it seems that there is a greater differentiation among organisms at transcriptome level than at genome level [87], maybe due to a higher evolutionary rate of the transcriptome [88], or to the epistatic and pleiotropic nature of the molecular mechanisms underlying gene expression [79]. On the other hand, the proteome level is the closest to the phenotype, and our previous results [54] showed higher differentiation between ecotypes at the proteome level, that is around 7%. Since the selection is acting at phenotypic level while variation is generated at the level of the genotype, the proportion of changes caused by selection can be expected to be largest at phenotypic level and smallest at the DNA sequence level [89], a view that seems consistent with the partial data we have available in the global analysis.

Another central, and yet controversial, question in evolutionary biology concerns the genetic basis of evolutionary change. King and Wilson (1975) [90] proposed that the key to understand the differences among species is not in the gene-coding, but in the DNA region that regulate the levels, locations, and time of gene expression. An important tenet of evolutionary developmental biology ("evo devo") is that cis-regulatory mutations are more important than structural mutations in phenotypic evolution [91], although it is also argued that adaptations likely involve a mixture of structural and cis-regulatory changes [92]. Here, the hypothesized increase of gene differentiation between ecotypes along the three different molecular levels could indicate a certain influence of the regulatory elements affecting to gene expression, but such hypothesis will need an independent corroboration. New sequencing effort will be necessary in order to improve our knowledge on the genetic architecture of adaptive traits in this model system.

## Conclusions

In this study we have improved our knowledge of the role of differential gene expression on the ecological adaptation of the marine snail *Littorina saxatilis*. Our results show that at least 4% of the sampled transcripts are being differentially expressed between ecotypes adapted to alternative habitats, highlighting important differences in gene expression in the face of gene flow. A candidate transcript of such ecotype differentiation was identified as Cytochrome c Oxidase Subunit I (COI), which is up-regulated in the exposed ecotype. Interestingly, COI is involved in the oxidative phosphorylation, suggesting an enhanced mitochondrial gene expression (or increased number of mitochondria) to improve energy supply in the ecotype subjected to the strongest wave action, in the same way that those genes identified at proteome level [54].

## Methods

### Sample collection and preparation

In July 2006, adult males and females of *L. saxatilis *were collected from Silleiro (NW Spain; 42°6' 17' 8''N; 8°53' 56'') in two different transects. RB and SU individuals were collected from the upper and lower shore levels of each transect respectively. The horizontal distance between the two transects was 50 m, whereas the vertical distance between the two ends of each transect was approximately 23 m. Eight biological replicates were sampled per shore level (which makes a total of 16 per ecotype when considering the two transects). Samples were collected simultaneously during 30 minutes and immediately transported to the laboratory, where snails were sexed according to the presence of penis in males and a brood pouch of shelled embryos in females, thus ensuring that individuals were sexually mature. Each biological replicate (of the RB or SU ecotype) contained a pool of 5 males and 5 females. Snails used for the qPCR analysis were collected from Silleiro in July 2009 using the same procedure. However, in this case, 9 biological replicates were used for each ecotype, each one including a pool of 5 males and 5 females.

### RNA extraction and cDNA synthesis

Prior to RNA extraction, shelled embryos of each female were discarded. Total RNA was isolated from pools containing 5 males and 5 females of the RB or SU ecotype using TRIZOL^® ^Reagent according to the manufacture's instructions. High quality starting RNA is essential for the cDNA-AFLP technique. Therefore, in order to assess the integrity of the total RNA, an aliquot of each sample was run on an agarose gel. Moreover, the concentration and purity (i.e. the A260/A280 ratio) of each RNA sample was checked with an UV spectrophotometer (UNICAM UV/Vis UV2). Next, the Turbo DNA-freeTM kit (Ambion) was used to remove any remaining contaminating DNA from the total RNA extractions. The concentration was measured again by spectrophotometry. Finally, cDNA was synthesized from 25 μg of total RNA using the SuperScriptTM Double-Stranded cDNA Synthesis Kit (Invitrogen) and a biotinylated oligo (dT)18 primer, following the conditions recommended by the manufacturer.

### cDNA-AFLP analysis

The cDNA-AFLP technique [9] was used with some modifications that are detailed in Martínez-Fernández *et al*. (2010)[11]. The restriction enzymes used were *Taq*I and *Mse*I, each with a 4 bp recognition sequence to obtain enough number of peaks by primer combination as in previous works with cDNA [93,94]. The digestion was performed in two separate steps. Briefly, 500 ng of cDNA was first digested with *Taq*I for 4 h at 37°C. Then, the enzyme was inactivated by heating for 10 min at 70°C and the 3' end fragments were collected on streptavidin magnetic beads by virtue of their biotinylated tails using the PolyATract^® ^Systems III kit (Promega). Next, cDNA fragments on the magnetic beads were digested with *Mse*I for 4 h at 37°C, followed by the inactivation of the enzyme for 4 h at 37°C. The supernatant, containing the digested fragments, was collected and used as template in the subsequent AFLP steps, while the 3'-end tails that remained bounded to the beads were discarded. In this way, the total number of tags to be screened was reduced, additionally favouring the collection of more informative tag derived at least partially from the coding region, and therefore facilitating functional characterization of the transcripts. *Mse*I and *Taq*I adaptors were ligated for 16 h at 16°C in a total volume of 50 μl. The *Mse*I and *Taq*I adaptors were prepared by mixing equimolar amounts (50pmol each) of the oligonucleotides 5'-GACGATGAGTCCTGAG-3' and 5'-TACTCAGGACTCAT-3' for *Mse*I adaptor and 5'-GACGATGAGTCCTGAG-3' and 5'-CGCTCAGGACTCAT-3' for *Taq*I adaptor. Pre-amplification of cDNA fragments was performed for 20 cycles with a 4 μl aliquot of a 1:10 dilution of the ligation reaction in a total volume of 20 μl, using 20 pmol of the primers corresponding to the *Mse*I and *Taq*I adaptor sequence without selective base (*Mse*I primer 5'-GATGAGTCCTGAGTAA-3', *Taq*I primer 5'-GATGAGTCCTGAGCGA-3'). Each PCR was performed at 94°C for 20 sec, 56°C for 30 sec, and 72°C for 2 min. The pre-amplified products were diluted 1:10 and a 4 μl aliquot was selectively amplified in a total volume of 20 μl with *Mse*I and *Taq*I primers having 1 selective base extension at the 3' end. Amplification included a touchdown phase for 10 cycles of PCR (94°C for 20 sec, 66°C for 30 sec, and 72°C for 2 min; annealing temperature being decreased 1°C every cycle), followed by 20 cycles (94°C for 20 sec, 56°C for 30 sec, and 72°C for 2 min). A total of 12 primer combinations were used for selective amplifications. *Taq*I selective primers were fluorescently labeled with different dyes (6-FAM, HEX and NED). The selectively amplified fragments were run on an ABI Prism 310 Genetic Analyzer with an internal ROX-labelled sizing ladder (Applied Biosystems).

AFLP profiles were visualized and analyzed using GeneMapper^® ^v.3.7 software (Applied Biosystems), within a fragment-length (size) range of 75-500 base pairs. To eliminate background noise, a DNA fragment was considered to be valid if it had a peak height of at least 50 relative fluorescent units (RFU) and a ± 2 base size difference with the nearest DNA fragment peak (transcript). Each AFLP expression profile was normalized using the sum of signal method as implemented in GeneMapper^® ^to correct for differences in total electropherogram intensities, which may arise due to loading errors or differences in amplification efficiency, being actually a semi-quantitative analysis. Moreover, all AFLP reactions were repeated twice (each reaction representing a technical replicate) to allow evaluation of the reproducibility of the method, which was calculated by the Pearson correlation coefficient for the quantitative data, and by the coefficient of similarity called "simple-matching coefficient" [55] for the presence/absence data. This coefficient gives the same biological importance to the coincidence in the presence or absence of a transcript. Moreover, it maximizes the amount of information drawn from an AFLP profile by considering all scored loci [95].

We used the whole set of 32 samples (16 biological samples from each ecotype, each one with 2 technical replicates) to choose exclusively those transcripts that were present in 90% of the replicates. In the randomization ANOVA, when a transcript was only detected in one of the two technical replicates, we assigned the threshold limit value of 50 RFU to the missing peak height, and used the average value of the two technical replicates.

### Statistical assessment of gene expression differences

Only common transcripts (present in at least 90% of the replicates) were considered in the comparison among groups. Quantitative transcript differences were assessed using a three-way ANOVA on normalized intensity data with the two fixed factors (ecotype and transect), the third factor (biological replicate) nested with the interaction, and the technical replicates as residual. This parametric three-way ANOVA was carried out with the SPSS/PC version 14. Here, the factor transect could be considered as a control of expression differences between ecotypes in the sense that any putative candidate loci of adaptation should produce significant differences in expression between ecotypes, but not significant differentiation between transects or interaction.

Significant cases were confirmed by a randomization ANOVA, using the freeware software ANOVA http://webs.uvigo.es/c03/webc03/XENETICA/XB2/software.htm, which is very robust to deviations from normality and heteroscedasticity (see [96]). The SGoF multitest adjustment [97] was used to correct for testing multiple hypotheses using the freeware software SGoF http://webs.uvigo.es/acraaj/SGoF.htm.

A hierarchical clustering was applied on the transcripts with significant quantitative differences in expression, using the uncentered correlation distance based on the Pearson correlation and the average linkage algorithm by the Cluster 3.0 and Java treeview software http://bonsai.ims.u-tokyo.ac.jp/~mdehoon/software/cluster/software.htm.

### Isolation and sequencing of transcripts

In order to isolate the peaks showing significant differences in expression, the PCR products of the corresponding selective primer combination were run in agarose gels to cut the bands corresponding to the appropriate peak size. cDNA was extracted from the gel piece using NucleoSpin^® ^Extract II kit (Clontech). To reamplify the differentially expressed transcripts, 1 μl of the eluted cDNA was amplified with the same selective primer combination in a 20 μl reaction volume. PCR conditions were an initial denaturation step at 95°C for 5 min, 30 cycles at 95°C for 30 sec, 56°C for 30 sec and 72°C for 30 sec, with a final extension step at 72°C for 10 min. The size of the amplified fragments was confirmed by capillary electrophoresis on an ABI Prism 310 Genetic Analyzer in order to test whether the isolated peak showed the expected size. The PCR product was purified and cloned using TOPO TA Cloning^® ^kit for Sequencing (Invitrogen). Finally several colonies from each PCR product were sequenced in order to determine if a single transcript had been effectively isolated from each peak, or whether if a peak contained more than one transcript type (homoplasy). Nucleotide sequences and translated sequences were compared with the nucleotide and protein sequences of the nonredundant Genbank databases and the sequences of the EST databases using BLAST and TBLAST sequence alignment programs respectively at the web server of NCBI in july 2010.

### Quantitative real-time PCR (qPCR)

For the qPCR analyses, total RNA was isolated from the pools using TRIZOL^® ^Reagent (Invitrogen, life Technologies) according to the manufacture's instructions. The integrity of the total RNA was assessed in the same way than for the cDNA-AFLP analysis. Genomic DNA was eliminated from the samples by a DNase treatment (Fermentas) according to the manufacturer's description, and its concentration was again determined by spectrophotometry. RNA from each sample (1 μg) was reverse transcribed in a final volume of 20 μl using the Transcriptor First Strand cDNA Synthesis kit (Roche Applied Science) and an oligo (dT)18 primer.

Specific primer pairs were designed for 6 candidate reference genes and 1 for the gene of interest Cytochrome c Oxidase subunit I (COI). Primer design was done using Primer 3 (v. 0.4.0; http://frodo.wi.mit.edu/primer3/. To ensure that fluorescent signals produced during qPCR assays represent only target amplicons, primer conditions were previously optimized by determining the optimal annealing temperature and primer concentrations to eliminate the formation of primer dimers that could contribute to fluorescence. Specificity of the amplicons was checked by running the conventional PCR products on 2% agarose gel to confirm the production of a single band of the predicted size. Moreover, a melting curve analysis was performed after every amplification program in the qPCR to verify specificity of the target and absence of primer dimers, besides of including a no-template control on every plate for each primer set to verify also that PCR master mixes were free of contamination.

Real time PCR was performed in the LightCycler 480 instrument (Roche Applied Science) with 20 μl reactions containing 10 μl of LightCyclern480 SYBR Green I Master, 7.6 μl or RNase free water, 0.2 μl of each primer (1 μM), and 2 μl of cDNA as PCR template. Cycling parameters were 95°C for 10 min to activate DNA polymerase followed by 35 cycles of 95°C for 10 s, 54°C for 15 s, and a final extension of 72°C for 9 s. Detection fluorescence was carried out at the end of each extension step. After amplification, a melting curve was acquired by heating to 95°C for 5 s, cooling to 70°C for 1 min, and slowly heating to 95°C with a continuous fluorescence data collection of 10 acquisitions per °C.

### Reference gene selection and statistical analysis

To determine the mRNA expression, stability of the 6 reference genes was measured using the qPCR as described above. Since the use of just a single reference gene may result in a more than 6-fold erroneous normalization [31], the geNorm software was used to decide the appropriated number of genes needed for a reliable normalization.

Raw quantification cycle (C_q_) values were transformed to relative quantities using the software program geNorm, version 3.4 [31]http://medgen.ugent.be/~jvdesomp/genorm/. The statistical algorithm geNorm derives a stability measure (M value) and via a stepwise exclusion of the least stable gene, creates a stability ranking. Genes with the lowest M values have the most stable expression, and therefore would be selected as ideal reference genes. In the iterative steps, genes with the lowest expression stability (i.e. the highest M value) are removed. A new M value for each of the remaining genes is calculated until only two genes remain. Because these calculations are based on ratios, the final 2 genes cannot be resolved.

In order to determine the differential expression of the gene of interest (COI), raw Cq values were transformed to relative quantities, normalized by the two best reference genes, using the software qBase version 1.2.2 (based on the formulas described in [98]). Then, the normalized values of expression were analyzed by a randomization one-way analysis of variance (ANOVA). To determine the reproducibility of the method, the same samples (9 RB and 9 SU) were run in different days and the correlation coefficient of Pearson was obtained. The probability of this coefficient was also calculated by randomization using Poptools software http://www.cse.csiro.au/poptools/. Once a good reproducibility was obtained, the confirmation of the differences in COI expression was assessed using the 9 biological replicates per ecotype. All other statistical analyses were performed with SPSS (v14, SPSS INc., Chicago, IL).

## Authors' contributions

MMF: carried out the molecular genetic studies, participated in the sequence alignment, drafted the manuscript, participated in the design of the study, and performed the statistical analysis; LB: participated in the study design and coordination, and helped to draft the manuscript; ERA & HQ: participated in the design of the study, performed the statistical analysis, conceived the study, participated in its design and coordination, and helped to draft the manuscript. All authors read and approved the final manuscript.
